# Patient safety – Development, implementation and evaluation of an interprofessional teaching concept

**DOI:** 10.3205/zma001221

**Published:** 2019-03-15

**Authors:** Katja Wipfler, Johanna Elisabeth Hoffmann, Anika Mitzkat, Cornelia Mahler, Susanne Frankenhauser

**Affiliations:** 1 Universitätsklinikum Heidelberg, Klinik für Anästhesiologie, Heidelberg, Germany; 2 Universitätsklinikum Heidelberg, Abteilung Allgemeinmedizin und Versorgungsforschung, Heidelberg, Germany; 3 Universitätsklinikum Tübingen, Abteilung Pflegewissenschaft, Institut für Gesundheitswissenschaften, Tübingen, Germany; 4 BG Unfallklinik Ludwigshafen, Centrum für interdisziplinäre Rettungs- und Notfallmedizin, Ludwigshafen, Germany

**Keywords:** Patient safety, risk management, interprofessional relations, interdisciplinary communication, professional education

## Abstract

**Objective: **Patient safety has high priority in health care. Since successful interprofessional collaboration is essential for patient safety, the topic should ideally be addressed interprofessionally in the curricula. The aim of the project was the development and implementation of an interprofessional teaching concept "patient safety" for medical students and students of health professions at the Medical Faculty Heidelberg.

**Methodology: **The learning objectives were formulated on the basis of the “Patient Safety Learning Objective Catalog” (“Lernzielkatalog Patientensicherheit”) of the Society for Medical Education (Gesellschaft für Medizinische Ausbildung, GMA) and on the basis of the American Interprofessional Competence Profile “Core Competencies for Interprofessional Collaborative Practice”. Two courses were designed for interprofessional groups of approximately 15 participants. The learning content was designed interactively through the development of the project, its application and critical discussion of error reporting systems and security checklists as well as role-plays and video material. The evaluation was carried out by means of descriptive analysis of a structured course evaluation system, which was developed for this study.

**Results: **28 students took part in the courses. 82% of the students considered the topic "patient safety" to be relevant. In 82% of the cases, the participants rated the interprofessional aspect of the course as valuable. Overall, 73% of students whished for more interprofessional education.

**Conclusion:** The results of the evaluation show that the teaching concept is well accepted by the students and encourage the implementation of further interprofessional courses with a thematic relevance.

## Zusammenfassung

**Zielsetzung: **Die Patientensicherheit besitzt einen hohen Stellenwert in der Gesundheitsversorgung. Durch interprofessionelle Zusammenarbeit kann die Patientensicherheit nachhaltig gefördert werden, daher sollte dieses Thema idealerweise in den Curricula auch interprofessionell gelehrt werden. Ziel des Projekts war es ein interprofessionelles Lehrkonzept "Patientensicherheit" für Medizinstudierende und Studierende der Gesundheitsberufe an der Medizinischen Fakultät Heidelberg zu entwickeln und zu implementieren.

**Methodik: **Die Lernziele wurden in Anlehnung an den „Lernzielkatalog Patientensicherheit“ der Gesellschaft für medizinische Ausbildung und auf Grundlage des amerikanischen interprofessionellen Kompetenzprofils „Core Competencies for Interprofessional Collaborative Practice“ formuliert. Anhand der Lernziele wurden zwei Lehrveranstaltungen für interprofessionelle Gruppen von ca. 15 Teilnehmenden konzipiert. Die Lerninhalte wurden interaktiv durch die Entwicklung, Anwendung und kritische Diskussion von Fehlermeldesystemen und Sicherheitschecklisten sowie anhand von Rollenspielen und Videomaterial erarbeitet. Die Evaluation erfolgte mittels deskriptiver Analyse eines selbst entwickelten strukturierten Kursbewertungsinstrumentes.

**Ergebnisse: **28 Studierende nahmen an den Lehrveranstaltungen teil. 82% der Studierenden hielten die Thematik "Patientensicherheit" für relevant. In 82% der Fälle bewerteten die Teilnehmenden den interprofessionellen Aspekt der Lehrveranstaltung als gewinnbringend. Insgesamt wünschten sich 73% der Studierenden mehr interprofessionelle Unterrichtseinheiten.

**Schlussfolgerung: **Die Ergebnisse der Evaluation zeigen, dass das Lehrkonzept von den Studierenden gut angenommen wird und ermutigen dazu weitere interprofessionelle Lehrveranstaltungen mit thematischem Bezug zu implementieren.

## 1. Introduction

### 1.1. Problem analysis

Patient safety has high priority in health care. Patient safety means on the one hand the absence of undesired events, on the other hand actions to avoid them [1]. The key message of the patient safety alliance is “patient safety is learnable!” [1]. So far, however, only little attention has been paid to the training of competences in this field [2]. According to the working group “Patient Safety and Error Management” (Ausschuss Patientensicherheit und Fehlermanagement) of the German Medical Education Society (Gesellschaft für Medizinische Ausbildung, GMA), complex diagnostic and therapeutic processes in healthcare also pose risks. Not only prevention, but also active constructive error management contributes to patient safety. In addition to sensitizing students and physicians to these issues, they should also be able to follow recommendations for specific practice. Openly dealing with organizational and communication deficits is an essential requirement [3]. The National Competence Based Catalogues of Learning Objectives for Undergraduate Medical Education (Nationaler Kompetenzbasierter Lernzielkatalog der Medizin, NKLM) has already created an approach to implement patient safety in medical curricula [4].

Patient care is a system of interdependent factors [1]. The idea “To err is human” is based on the fact that mistakes are not usually made by bad people, but due to lack of improvement in security structures. “The problem is not bad people; the problem is that the system needs to be made safer.” [5]. A change in perspective is fundamental to patient safety, alongside the recognition of system causes of errors and factors contributing to critical events. This is imperative to be able to develop and implement adequate strategies to avoid such events [1]. 

So far, the training of medical students and trainees in the health professions is predominantly monoprofessional [6]. However, the changes in the health system are changing the fields of action of all health professionals placing new demands on their qualifications. Interprofessional learning is gaining importance in order to improve interprofessional collaboration and patient care [7], [8], [9]. Interprofessional collaboration is considered a key competence in many areas of healthcare [10], [11]. The WHO strongly recommends integrating this competence as an integral part of training into the curricula of the individual health professions [12]. Interprofessional learning is defined as learning from, with and about each other of two or more professions promoting collaboration and quality in health care delivery [7]. The discrepancy between required and actually taught competencies was the starting point to develop interprofessional teaching units on patient safety at the Medical Faculty of Heidelberg.

#### 1.2. Solution strategy

In addition to continuous quality assurance, interprofessional and cross-sectoral strategies for error prevention, as an important aspect of patient safety, are becoming increasingly important [13]. Patient safety can only be sustained through effective collaboration between all actors involved in patient care [14]. Thus, the Alliance “patient safety” (Aktionsbündnis Patientensicherheit) recommends that relevant topics are to be addressed already in the curricula of all health professions [1].

Interprofessional collaboration seems to be essential in order to improve thinking and actions across professional groups, thereby contributing to better patient care [8]. The promotion of interprofessionality can be formulated as a goal of competence-oriented teaching. In the field of medical education, corresponding courses must be developed, implemented and consolidated in the long term [8], [11]. On a national level, there are already approaches to establish interprofessional courses, among other topics emergency training and ward round simulations are addressed [6], on an international level Reeves et. al. for example give an overview on interprofessional education in health professions [15], [16], [17], [18].

At the Medical Faculty of Heidelberg, in addition to the medical and dentistry programmes, the bachelordegree Interprofessional Health Care (IHC) has been offered since 2011. In this programme, students of nursing, therapy and diagnostic professions complete a vocational training in parallel to their university studies obtaining a bachelor's degree. The chosen name of the degree program implies the cross-professional orientation and the curriculum provides sufficient scope to implement innovative interprofessional courses [19]. Initial approaches to bidirectional integration have been implemented since 2012 [20]. The “Masterplan 2020” envisages that in addition to the acquisition of professional competences, the curriculum of the medical programme will focus on the promotion of collaboration, mutual acquaintance and the development of a common understanding of the future working life [21].

#### 1.3. Objective of the project

The aim of this project was the development of an interprofessional teaching concept on “patient safety” at the Medical Faculty of Heidelberg. In joint courses medical students in their last year of study (“practical year”, Praktisches Jahr, PJ) are taught together with students of the bachelor programme Interprofessional Health Care (IHC). The teaching concept assumes that involving several professional groups in health care delivery opens a more complex approach to the topic than a monoprofessional approach. The sessions are interactive with alternating work in small groups and plenum. A role-playing game and case studies which were designed for this course are of particular importance. Additionally, a best practice video was developed to visualize possible failures in health care delivery associated with suboptimal interprofessional collaboration.

## 2. Project description

### 2.1. Curricular anchoring

Patient safety is a subject in various courses at the Medical Faculty Heidelberg but not yet in form of a practice-oriented interprofessional teaching concept. The presented new interprofessional seminar is mandatory for medical students, anchored in their four-month deployment in anesthesiology and mandatory for students of Interprofessional Health Care, anchored in their seventh semester of study (Modul 8 “Interprofessional Health Care”). A maximum of 15 participants (7-8 medical and IHC-students, respectively) take part in the session providing the opportunity to gain insights into the topic from the perspective of multiple health professions and jointly considering a way to deal with errors in healthcare.

#### 2.2. Learning goals

The learning objectives of the curriculum relate to the acquisition of professional competencies, moreover interprofessional competencies should be addressed. The professional competencies were formulated in accordance with the recommendations of the GMA learning objective catalog “patient safety” [22], the interprofessional competencies based on the American interprofessional competence profile “Core Competencies for Interprofessional Collaborative Practice” [23]. The main focus and aim within this course was to develop students’ “Interprofessional Collaborative Competencies”.

A learning objective catalogue for the course was developed specifically for this seminar. Exemplary learning goals are shown in table 1 (Tab. 1).

#### 2.3. Design and structuring

The interprofessional courses took place for the first time in the summer semester 2017 and have been held twice a year since then. 

Not only the participants were mixed interprofessionally (students of the medical programme and Interprofessional Health Care) but also an interprofessional faculty tandem (physician and nurse) was built bringing together both perspectives. The development and implementation were regularly discussed and reflected in an interprofessional and cross-institutional project group. 

Various teaching methods have been utilized for course implementation, focusing on interprofessional exchange, the promotion of collaboration and elimination of barriers. The first lesson includes the topic “Critical Incident Reporting System” (CIRS), the professional input is given as an introductory lecture. The main objectives are getting to know the different professions involved in health care, a role play, small group work and case studies.

The second lesson highlights the methods implemented for a sustainable promotion of patient safety. The professional input is analogous to the first appointment held as an introductory lecture. Afterwards, the students work on their own safety checklists in small groups, discussing influencing factors of patient safety and reflecting tasks and responsibilities of their own or other professions using a teaching video.

In the final teaching unit, the student's attitude regarding patient safety and interprofessional collaboration will be discussed. There is no graded test required for the course. Nevertheless, in order to reflect and record the objectives of the course the students are requested to write a“„one-minute-paper” summarizing the gain in their personal knowledge and competencies. The detailed schedule of the course is shown in table 2 (Tab. 2). 

#### 2.4. Didactic approach

The didactic concept is based on the concept of “experience based learning” by Dewey, according to which increased knowledge is achieved only by practical approach and reflection [24].

##### 2.4.1. Role play

The didactical decision for a role play was made for obvious reasons, which include the advancement of practical skills and to step into a real observer position. Additionally, communication and problem solving competencies are supported. 

The role play addresses as many involved professions as possible. A clinical setting was selected which seems to be transferable to other fields of health care dealing with a general problem applicable to all professions. 

Essentially, the role play addresses a patient file being accedentialy swaped in an outpatient surgical setting. A number of patients are to be treated sequentially however according to the Swiss cheese model by Reason [25] similar names and lack of safety checks at different stages result in the risk of performing surgery on the wrong patient. The role play is performed by seven actors, the remaining students act as critical observers of the situation. After performing the role play, the individual steps are summarized by the external observers and all participants then work out the intended learning goals.

##### 2.4.2. Case studies

The key component of a clinical risk management system is a feedback system for critical events (CIRS), in which so-called “nearly damage” is to be recorded and analysed. The term “nearly-damage” refers to errors or events that could have been harmful for the patient, but were not the specific case [26].

In order to give the students the opportunity to apply acquired knowledge in different interprofessional settings, given case studies are developped in small groups and then discussed in plenary sessions. During discussion, various levels of competence are examined based on the Miller pyramid [27].

##### 2.4.3. Best practice video

The idea of developing a best practice video for teaching purpose was that many students might be unfamiliar with perioperative procedures. Since the perioperative environment is prone to errors, it is suitable for teaching.

In order to show the importance of the involvement of the various professional groups, effective collaboration and good team communication, a patient-actor was accompanied in an approximately 10-minute video starting with assessment on the ward and finally in the recovery room. The students see all the different stages of perioperative care. The students are asked to actively listen and critically evaluate the procedure, focusing especially on the responsibilities of the involved professions and on actions that might pose a risk to the patient. 

#### 2.5. Evaluation

To evaluate the project, a structured course assessment tool was developed. It covers the topics “relevance for later professional life”, “knowledge gain”, “attitudes towards the handling of patient safety” and “interest in interprofessional education”. Additionally, a free-text comment was possible (“I particularly liked the course because…”/ “To improve the course, one should…”) The questionnaire consists of five closed and one open questions, four of which were Likert-scaled and one was designed as “forced choice”. The aim of the evaluation in the pilot phase was to record the subjective assessment of students in terms of knowledge acquisition on the topic patient safety. Furthermore, an assessment of whether the students accepted and benefited from the interprofessional course is carried out and if the wish for more such interprofessional lessons was present. Questions with professional content (relevance of the topic for later professional practice, knowledge gain and attitude) were Likert-scaled. The question of the assessment of the individual gain resulting from the interprofessional composition of the course (question 4) was emphasized. At the end of the course there was time for a short oral feedback, which was logged by the lecturers. The statistical analysis was carried out using SPSS version 21.0 (SPSS Inc., Chicago, IL, USA). The discriminant analysis was applied.

## 3. Results

The interprofessional course was carried out as a pilot project with a total of 28 students in the summer semester 2017 and the winter semester 2017/18. Great effort was necessary for the planning and coordination of the curricula, since they were integrated in two different study programmes. Both pilot runs were held by the same lecturers. The students completed the 2x90-minute courses on patient safety, which were conducted by the same lecturers. Participation was mandatory for both student groups. All students received an evaluation form immediately after participating. The return of the questionnaires was 89%. The average age of students was 25 years (min: 21, max: 34, SD 2.75) and 68% of the participants were female. All students already had practical experience. An overview of the sociodemographic information of the participants is presented in table 3 (Tab. 3).

**Figure 1:**
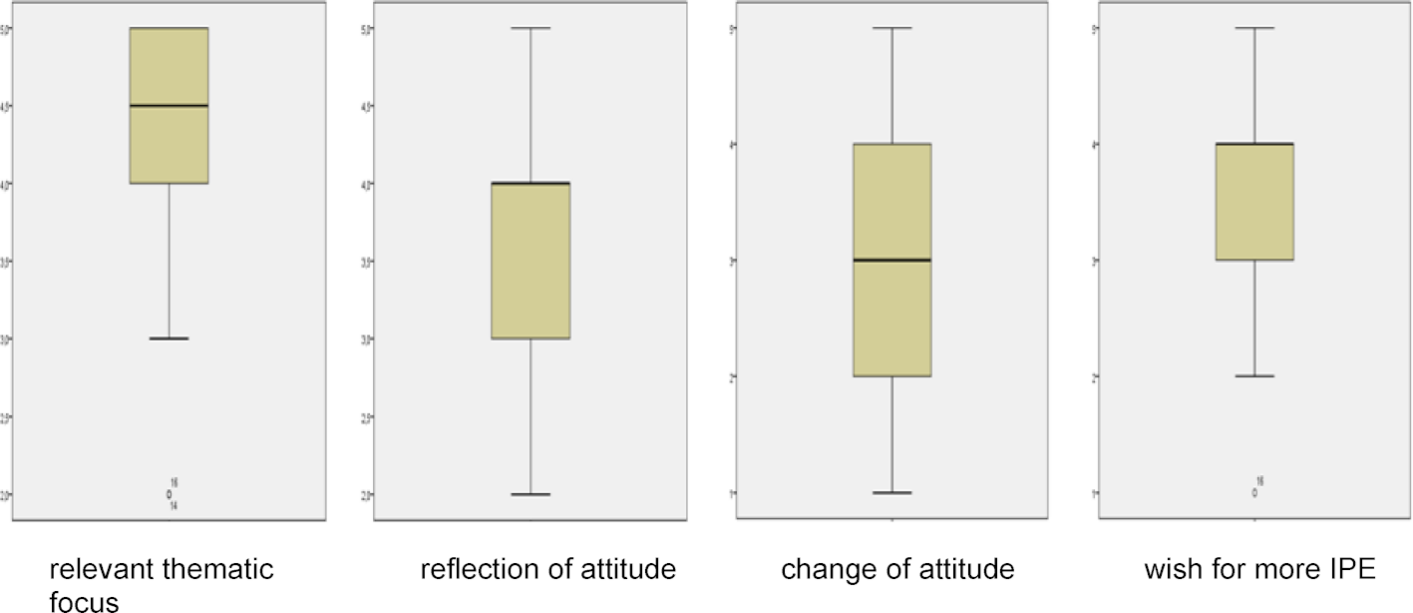
Percentage response distribution of Likert-scaled questions shown as box plot

As shown in table 4 (Tab. 4) and figure 1 (Fig. 1), 81.8% of the students consider patient safety (question 1) to be a relevant topic. 68.2% were encouraged to reflect on their own approach to patient safety and error culture (question 2). A change in attitude towards patient safety and error culture was reported by 36.4% of students (question 3). In 81.8% of the cases the participants were able to benefit from the interprofessional composition of the course (question 4). Overall, 72.7% of participants wished for more interprofessional education. 

Open text comments were provided by 11 students. A total of 14 comments were made, including 11 positive and 3 negative. The positive aspects can be summarized in three categories: group (size, atmosphere, cooperation), which was mentioned 4 times, interprofessionality was mentioned 4 times and the topic patient safety 2 times. One criticism states that medical and nursing aspects are emphasized (“the courses are mainly directed toward medical and nursing students”). This can be attributed to the chosen perioperative setting. One comment criticized the length of the teaching session and another the length of group work (“one too long group work”). No further details were stated by the evaluators.

From the lecturer’s perspective it was noticeable that the students of the different programmes (medicine and interprofessional health care) were not familiar with each other at the beginning of the course. Unfamiliarity with the other profession could be reduced during the course. A lively interprofessional discussion of the topic was achieved after the reduction of prejudice and tension.

Despite the limited time, the students generated further fields of interprofessional teaching opportunities for patient safety such as transfusion, hygiene, handover or handover simulations. As an oral feedback, the students mentioned i.a. that it is important to learn about the tasks and responsibilities of other professions (“There is more need to enhance the knowledge of other professions and their tasks and responsibilities during the training phase”) and that the topic had not been addressed in the curricula so far (“the subject of patient safety has so far been poorly reflected in the existing teaching concepts”).

## 4. Discussion and perspectives

### 4.1. Evidence and limitations

The results show that the participating students are interested in dealing with the subject of patient safety in an interprofessional course. The interpretation of the results is strongly limited as the sample of 28 students is not representative. Nevertheless, the results of the pilot stage show a tendency that is important for further local development. The data should be supplemented with data from other cohorts for more reliable interpretations. The interprofessional implementation of the course followed the assumption that interprofessional collaboration improves quality of care [28]. 

The question arises if, in principle, interprofessional education is more suited to address topics like patient safety or if similar results can be achieved with monoprofessional courses. 

An outpatient perioperative setting was chosen for the course. The topic of patient safety can also be discussed using other settings. The students are given enough time during group discussions to transfer the knowledge into their profession. The goal is to empower students to independently engage with patient safety in other areas of healthcare. However, one should bear in mind that interprofessional collaboration within the course may not easily be transferable to other domains [29].

#### 4.2. Quality assurance and consistency

The development, implementation and continuity of new teaching concepts are generally time-consuming and accompanied by numerous challenges. The implementation of the interprofessional teaching concept requires the adaptation of the curricula of two previously independent courses, which would facilitate joint instructions [11]. To serve as a role model for the students, all courses are taught by an interprofessional faculty team. Thus, the conception, design and implementation of the course are associated with relatively high human resources. For a successful and long-term conception, implementation and stabilization of the teaching concept, structural, personal as well as financial barriers have to be overcome.

The decision to establish the teaching concept at a relatively late stage in professional training is due to the fact that one's own work experience is a basic prerequisite for an emerging discussion and that professional barriers according to the lecturers were considered rather low at that time. According to the WHO, an interprofessional and interdisciplinary approach is essential to ensure optimal patient safety and is best practiced both theoretically and practically in education [12]. It would be conceivable to separate theory and practice. Theoretical courses could be established profession-specific in an early phase of education and practical lessons towards the end of training. The evaluation of competence acquisition is currently not measurable in the presented education concept. Students' interprofessional teamwork during class, such as discussing CIRS cases or creating a checklist, can only indicate the success of the learning objectives. In order to evaluate the actual acquisition of competence, a suitable examination format would have to be introduced in the sense of the Constructive Alignment. A revision and further development of the teaching concept with introduction of a final examination are conceivable, whereas the review of attitudes regarding the subject patient safety remains challenging [30]. In order to ensure the long term teaching quality, joint planning including the institutional, professional and status factors as well as the further analysis of the evaluation data are of great importance. Further goals are the adaptation as well as a curricular stabilization of the courses in both programmes.

As mentioned in 4.1, the perioperative setting does not cover all aspects of patient safety. It is important to identify further fields of health care and also to establish courses on patient safety in other disciplines. An interdisciplinary and interprofessional approach seems to be necessary in order to provide the students with additional learning objectives of the GMA “National Competence Based Catalogues of Learning Objectives for Undergraduate Medical Education” [22]. For the expansion of the education program, further cooperation with other disciplines of the University Hospital Heidelberg is already being planned.

## 5. Conclusion

Patient safety can be considered as an essential topic within interprofessional education. Despite the described barriers, the implementation of the pilot project was able to be put into practice and the teaching concept was well received by the students of the Medical Faculty Heidelberg. The results of the evaluation and the subjective impressions of the lecturers show a tendency that students benefit from the interprofessional courses. In future, the development of a reasonable competence based assessment and an evaluation using a validated assessment, such as the “University of Western England Interprofessional Questionnaire”, are pending.

## Acknowledgements

The authors thank Dr. med. Christopher Neuhaus for the support of the lectures and the introduction of his professional expertise on patient safety.

## Competing interests

The authors declare that they have no competing interests. 

## Figures and Tables

**Table 1:**
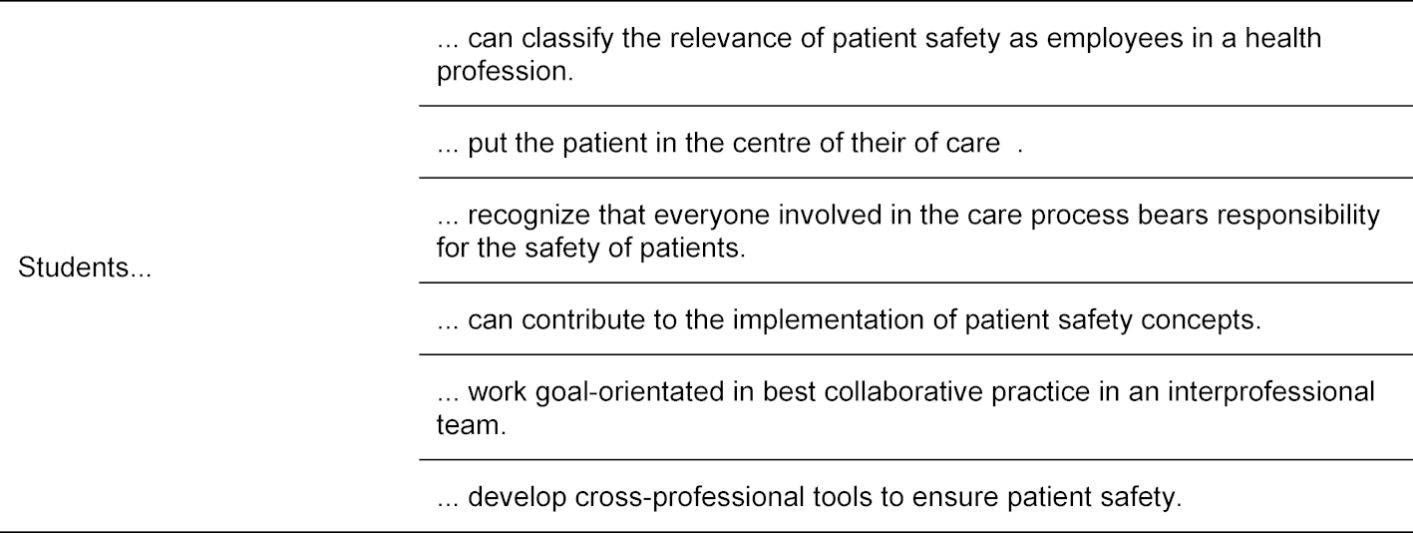
learning goals of the course

**Table 2:**
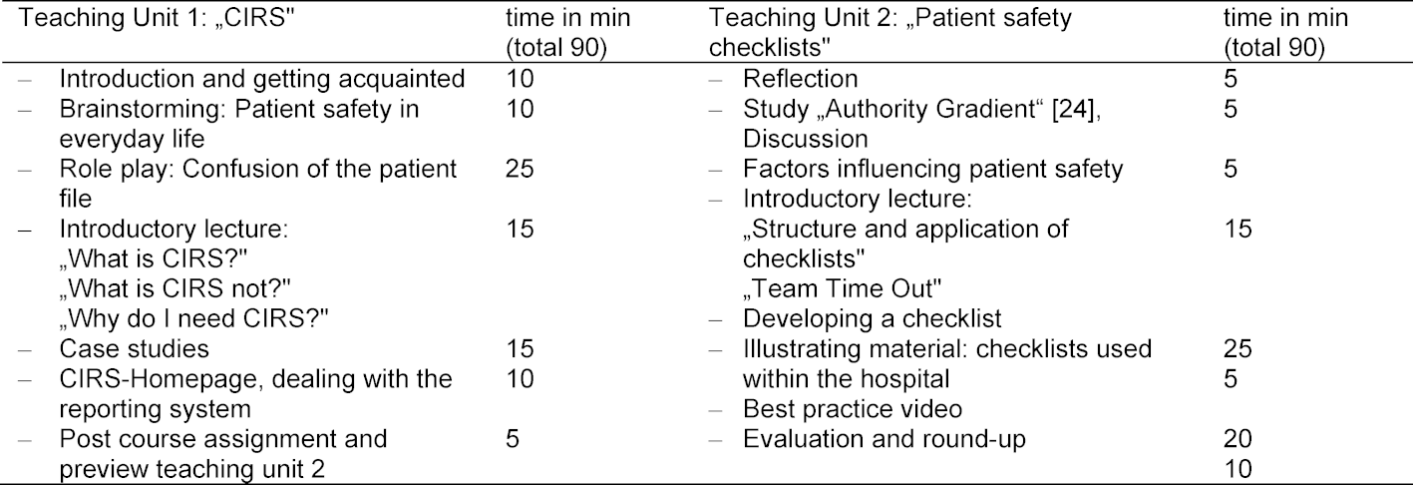
Patient safety schedule

**Table 3:**
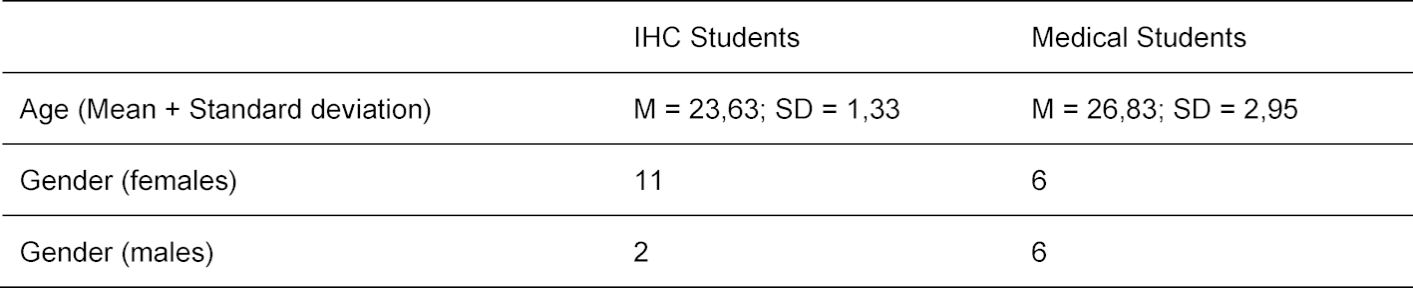
Sociodemographic information of participants

**Table 4:**
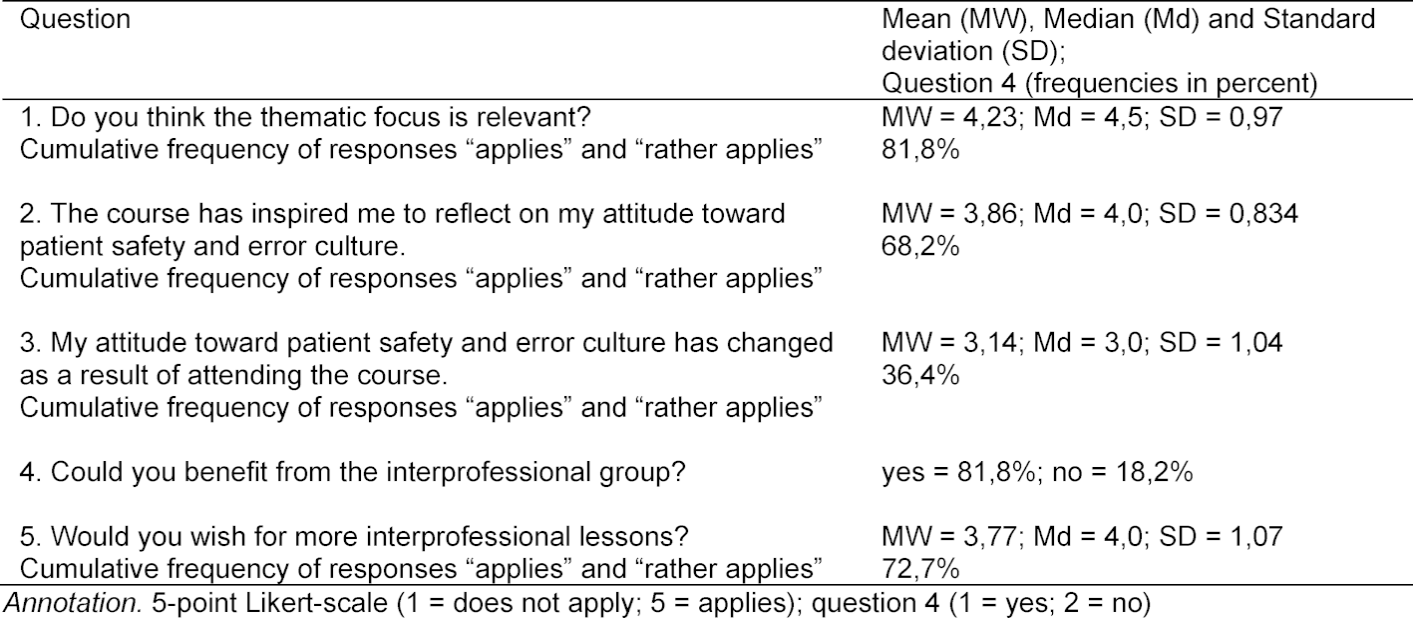
Evaluation of the course (Own course evaluation instrument)
